# Ultrasonically tuned surface tension and nano-film formation of aqueous ZnO nanofluids

**DOI:** 10.1016/j.ultsonch.2020.105424

**Published:** 2020-12-24

**Authors:** Elif Begum Elcioglu, S.M. Sohel Murshed

**Affiliations:** a Department of Mechanical Engineering, Faculty of Engineering, Eskisehir Technical University, 26555 Eskisehir, Turkey; b Center for Innovation, Technology and Policy Research (IN+), Department of Mechanical Engineering, Instituto Superior Técnico, University of Lisbon, 1049-001 Lisbon, Portugal

**Keywords:** Nanofluids, Surface tension, Ultrasonication, Nano-film, Statistical analysis

## Abstract

Nanofluids’ thermophysical properties and heat transfer performance has been investigated for many years, while research on their surface tension (ST) and wetting behavior is very limited. To assess nanofluids potential as industrial products, a complete picture is required to prove their performance in a specific application. Boiling heat transfer, microfluidics and drug development are among the applications where ST is a variable. ST of water-based ZnO nanofluids were measured in the presence and absence of direct ultrasonication. The experiments covered variation of ST with ZnO concentration (0.05–0.4 vol%), ultrasonication amplitude (40% and 100%) and duration. To the best of the authors’ knowledge, this is the first report of ST– ultrasonication process relation for a nanofluid. Results showed that after direct ultrasonication, nanofluids ST is strongly affected by the temperature raise, and in those cases relative ST may provide a clearer picture. A nano-film over individual and agglomerated nanoparticles spotted via TEM imaging was affected from the ultrasonication. Such a nano-film can play a key role in the anomalous thermal transport and wettability of nanofluids. Statistical analyses revealed that changes in ultrasonication amplitude resulted in a statistically significance difference on nanofluid ST and relative ST. Changes in nanoparticle concentration caused a significant difference on the nanofluid ST while the difference in relative ST was insignificant. Variation of ultrasonication duration caused significant variations on the relative ST while the difference in nanofluid ST was not significant. This work highlights that based on specific applications ST and other related features of any nanofluid can be adjusted employing proper ultrasonication conditions.

## Highlights

• The first report of ultrasonically-tuned surface tension (ST) of a nanofluid.
• Weak *ST_nf_ -*amplitude correlation and moderate *ST_r_-*amplitude correlation observed.
• Weak *ST_nf_* -duration correlation and strong *ST_r_-*duration correlation observed.
• Weak-to-moderate *ST_nf_*-volume fraction correlation observed.
• Presence of nano-film for alteration of surface and wetting behavior of nanofluid.

## Graphical abstract

**Figure:**
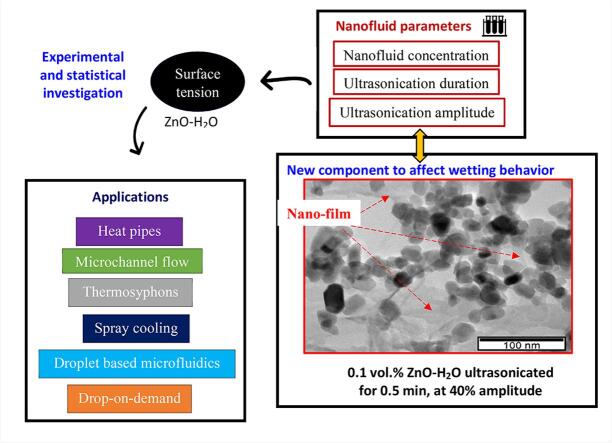

## 1 Introduction

Nanofluids has attracted immense attention from researchers worldwide [1] while their thermophysical properties and heat transfer performance has been under extensive research during the past decade. Such a high research interest is mainly due to the reported (earlier and current) improved thermophysical properties and heat transfer coefficients compared to base liquids with poor thermal characteristics, and for nanofluids potential in thermal management applications. Besides the mentioned improvements and cooling applications, nanofluids have found so far research-based application areas including solar collectors ([2], [3], [4], [5]), photovoltaics [6], and enhanced oil recovery [7], to name a few. While research on thermal properties of nanofluids has remained the focus of most of the studies, research on surface tension and wetting behavior of this new generation fluids is very limited. However, to assess nanofluids potential as industrial products, a complete framework is required to prove their performance in a specific application. Although thermal properties have utmost importance for heat transfer applications, surface phenomena always play an important role for their outstanding properties. Among surface features, surface tension (ST) is present when there is a density discontinuity between adjacent phases (e.g., liquid/air). As the miniaturization trend proceeds for development of advanced devices with characteristic dimensions at sub-micrometer scale, importance of surface properties increases, resulting in diminishing inertia effects and increasing ST [8]. ST plays key role as a variable in numerous thermal techniques and applications such as boiling [9], two phase flow in microchannels, heat pipes, thermosyphons [10], droplet-based microfluidics [11], and it determines the shape of the droplet [12]. ZnO nanofluids have gained attention due to outstanding thermal, semiconductor, and biomedical properties of ZnO nanoparticles. Adil et al. [13] studied 0.1 wt% ZnO-brine water nanofluids prepared by two-step method where they synthesized ZnO nanoparticles via sol–gel method and produced nanoparticles of 55 nm average size. They also used SDS, SDBS, OA as stabilizers and employed NaCl to obtain a brine from deionized water (DIW) as the base fluid. In this system, a pH value of 7.37 was determined to be the point of zero charge, at which the zeta potential was zero. Their results showed that SDS provided the lowest stability while SDBS provided the highest. Chung et al. [14] prepared dispersions of ZnO nanoparticles purchased from two different companies in DIW and used ammonium polymethacrylate as the dispersant. Their sedimentation study showed that the effective nanoparticle volume fraction over time was the highest when ultrasonic horn was used and was the lowest for the samples prepared using magnetic stir only. Kole and Dey [15] prepared ZnO-ethylene glycol (EG) nanofluids of 0.5–3.75 vol%, without surfactant, and used prolonged direct ultrasonication durations (>60 h). They reported an optimum ultrasonication duration (60 h) providing the highest nanofluid thermal conductivity for 3.75 vol% sample, beyond which (>60 h) thermal conductivity started decreasing. As mentioned before, ST is an important property in a variety of applications, including heat transfer which has been the main area that nanofluids hold potential in. Chinnam et al. [16] measured the ST of propylene glycol (60%) and water (40%) based Al_2_O_3_, ZnO, TiO_2_ and SiO_2_ nanofluids for varying temperatures (30-70℃), nanoparticle concentrations (<6 vol%), and nanoparticle sizes (15–30 nm). They reported that at a fixed nanoparticle concentration, ST of nanofluids decreased with temperature. For ZnO nanofluids, the ST was negatively correlated to particle size within 36–50 nm. All samples showed decrease of ST with increased nanoparticle concentration. Harikrishnan et al. [17] studied DIW, EG, and glycerol based CuO, Al_2_O_3_, Bi_2_O_3_, ZnO, and MgO (all with different morphologies) nanofluids ST, at constant temperature and in the presence of SDS, CTAB, and DTAB as surfactants. They concluded that particle morphology and available surface-to-volume ratio were effective parameters on ST. When dispersed in DIW, the ST values were the highest for Bi_2_O_3_ nanofluids, followed respectively by ZnO, CuO, and Al_2_O_3_. Bhuiyan et al. [18] measured ST of distilled water (DW) based Al_2_O_3_, TiO_2_, and SiO_2_ nanofluids. Their results revealed that ST increased as the nanoparticle concentration increased, while TiO_2_ nanofluids ST was greater than those of Al_2_O_3_ and SiO_2_ nanofluids’ due to nanoparticles bulk density variations. Radiom et al. [19] characterized TiO_2_-water nanofluids contact angle and ST. They reported that ST was not dominantly affected by nanoparticle concentration unless it was high (2 vol%). Soleimani et al. [20] studied ST of water based ZnO nanofluids in the presence of SDS as a surfactant, for oil recovery applications. They reported an increase in ST when the nanoparticle concentration increased from 0.05 to 0.3 wt%, followed by a sharp decrease at 0.4 and 0.5 wt% (the latter was attributed to the increased amount of SDS in the suspensions as the nanoparticle concentrations increased). Tanvir and Qiao [21] studied ST of ethanol, n-decane, and water based Al_2_O_3_, Al, B, and MWCNT nanofluids of 0.1–10 wt% concentration. DIW based MWCNT and Al_2_O_3_ nanofluids ST showed little change with nanoparticle concentration until 4 wt%, and then it increased for higher concentrations. For ethanol based nanofluids, increases and decreases in ST’s did not follow a straightforward trend for Al_2_O_3_, Al, B, and MWCNT nanofluids. Huminic et al. [22] studied FeC-water nanofluids ST. Their results showed that ST increased with FeC nanoparticle concentration within (0.1–1 wt%). Between 10 and 70 ℃, ST of water was greater than those of 0.1 wt% and 0.5 wt% nanofluid, while it was lower than that of 1 wt% nanofluid. Wanic et al. [23] measured the ST of EG based AlN, Si_3_N_4_, and TiN nanofluids, containing nanoparticles of different sizes, via du Noüy ring method and pendant drop method. They reported that particle size, surface area, morphology, and nanoparticle concentration did not have a considerable effect on the ST of studied nanofluids.

As summarized above, nanofluids ST dependences on adjustable nanofluid parameters (such as nanoparticle concentration, size, and preparation conditions) are at the developing stage. Ultrasonication has been shown to be a critically important stage both in preparation of nanofluids as well as re-sustaining their colloidal stability. It is an essential process for nanofluids prepared by two-step method. To the best of the authors’ knowledge and literature review, ultrasonication parameters dependence of nanofluids ST has not been reported yet. This topic is considered to be fundamentally important, since via ultrasonication thermophysical properties of nanofluids as well as dispersion and size distribution of nanoparticles can be improved and tuned. Ultrasonication parameters effect is hence important to fine-tune the ST as the application necessitates. This work is focused on ST measurements and statistical analyses on ST and relative ST of ZnO-water nanofluids of varying concentrations (0.05–0.4 vol%) that were treated ultrasonically at different amplitudes (40% and 100%) for different durations (0.5 min to 4 min). The ST results are supported by TEM imaging and statistical analyses to conclude realistically on the dependences of ST on these parameters. This work will provide a surface phenomenon-based information for ZnO-based thermal applications, as well as biomedical applications with ZnO nanoparticles.

## 2 Material and methodology

ZnO nanoparticles at different concentrations were used for preparing the aqueous (distilled water (DW)) nanofluids. It is to be noted that the purchased DW used as base fluid are pure, free of salts, metallic ions and oxidizers. The material preparation details, and investigation methodology are provided herein.

### 2.1 Nanoparticles morphological characterization

**Fig. 1:**
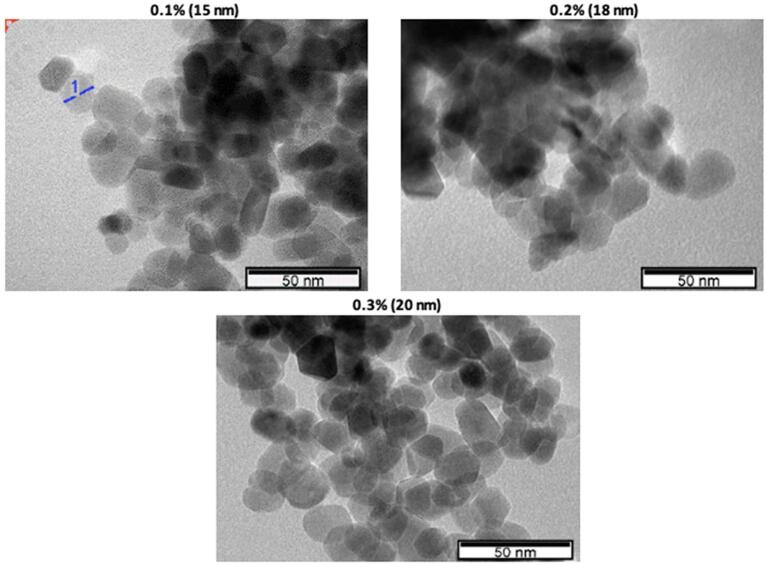
TEM images of ultrasonically untreated ZnO-DW nanofluids.

Nanofluids characteristics’ dependence on nanoparticle properties, morphology and state has been reported in the literature. Since surface properties’ importance is amplified for nanoparticles compared to larger solids, it is important to show the link between their presence and nanofluids properties. ZnO nanopowders (i.e., Zn (II)oxide powders) used in this study were obtained from IoLiTec Nanomaterials, Germany. The primary average particle size and shape provided by the manufacturer were 20 nm and spherical. However, a Transmission Electron Microscopy (TEM) imaging study was performed for the morphological characterization of nanoparticles. Sizes of the particles are measured during imaging from the representative regions on the images. From TEM images analysis of nanofluids at different concentrations (Fig. 1) it was found that the primary particle sizes are close to the value provided by the manufacturer (20 nm). The images in Fig. 1 show that the sizes of nanoparticles are around 15–20 nm. Also, TEM images demonstrate that the nanoparticles have nearly spherical or in polygonal shape.

**Fig. 2:**
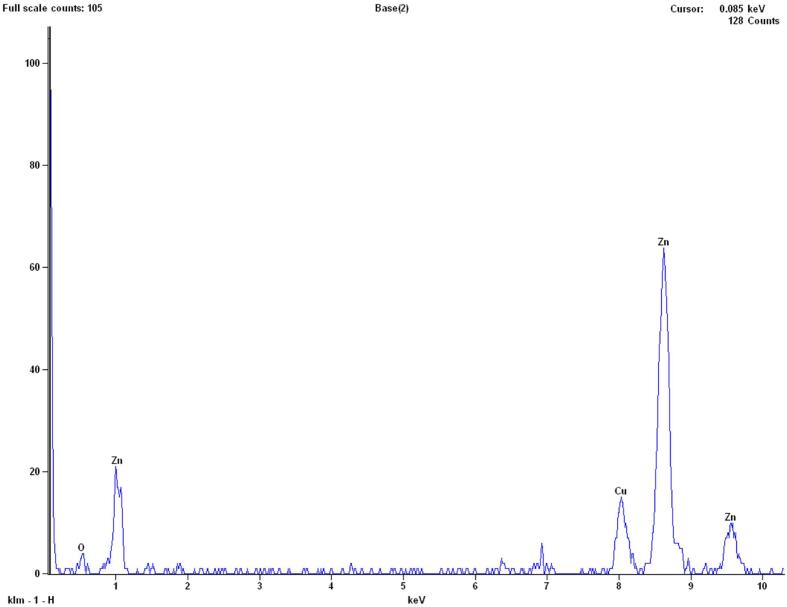
EDS results of nanofluid at 0.2 vol% of ZnO nanoparticles.

An Energy Dispersive Spectroscopy (EDS) analysis was performed to determine the purity and chemical composition of this nanoparticles. The EDS results of 0.2 vol% ZnO-DW nanofluid is presented in Fig. 2. It is noted that this sample nanofluid was not ultrasonicated since EDS is concerned with elemental information. As can be seen from Fig. 2, the peaks correspond to the presence of Zn and O from the ZnO nanoparticles and Cu from the grid used as the holder. Although the peaks of O molecules are small, they appeared at high quantity (%). No other significant elemental or compositional presences are detected.

### 2.2 Preparation of nanofluids

The nanofluids were prepared by the two-step method. The necessary amount of ZnO nano-powders was added in 25 mL of DW to prepare 0.5–4 vol% nanofluids. ZnO nanoparticles were weighed by an analytical balance (KERN ABS 80–4 N), with 0.1 mg readability and 80 g weighing range. After the nanoparticles and DW were mixed, the solution was stirred using a magnetic stirrer (ROTILABO® MH 15) for up to 30 min to enhance nanoparticle-base fluid mixing. During this stage sample was not heated. Stirring is the last stage of the preparation of samples that are not ultrasonically treated. Besides these samples, their ultrasonicated versions were prepared, which were treated at either of two ultrasonication amplitudes (40%, 100%) for different ultrasonication durations (0.5, 1, 2, 3, 4 min.) via direct ultrasonication (Hielscher UP200Ht). During ultrasonication, the amplitude and duration was controlled by the software, while the device adjusted the ultrasonication power accordingly during the process. Thus, ultrasonication parameters of this work are ultrasonication amplitude, and ultrasonication duration.

### 2.3 Methodology

Following the nanofluid sample preparation and ultrasonication, the samples were investigated by means of the following techniques and approaches: (i) TEM imaging and EDS analysis for particle morphology and chemical composition check, (ii) ST measurements, (iii) statistical assessment of the collected ST data.

**Fig. 3:**
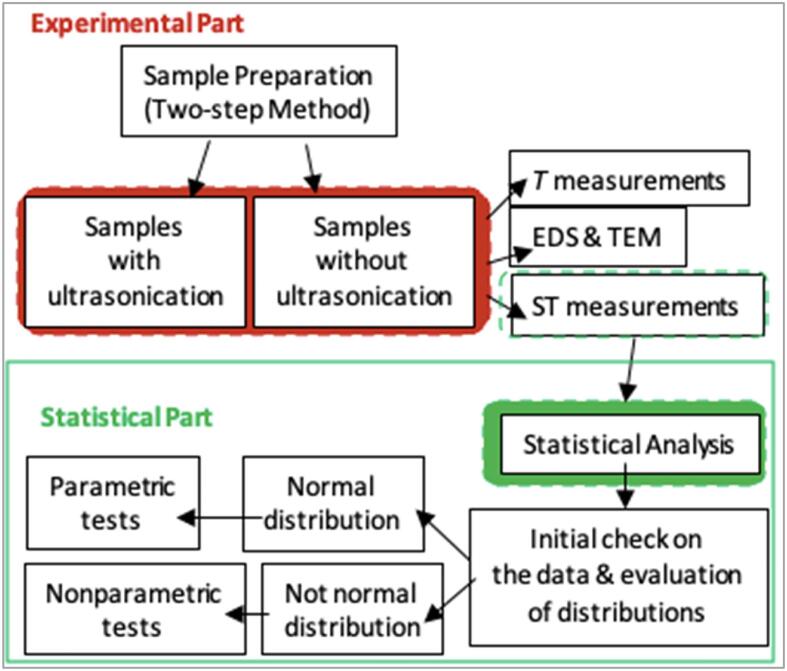
Schematic presentation of the methodology of this work.

ST measurements were performed using an Attension Theta Optical Tensiometer. The analysis mode during ST measurements was Young-Laplace. During ST data collection in each experiment, the drop-out sample size was 5 µL. The device records images for 10 s after the droplet is out of the dispenser. All measurements were conducted at room temperature, although some of the samples’ temperature (*T*) had risen by the ultrasonication process. The ST of ultrasonicated samples were measured right after they were taken out of the ultrasonicator, as ZnO nanoparticles’ dispersion in DW is far from stable, and controlling temperature externally or waiting for the samples to cool down would create a bias in ST data - ultrasonication duration relation. From applications point of view, if ZnO-DW nanofluids are to be used in a process where ST is important, and they are imperative to be ultrasonicated, then temperature rise is an inevitable consequence, so as the differences in particle–particle interaction (especially the Brownian motion) affected by the temperature rise. On the other hand, ST – *T* dependence is strong for most of the known liquids. In order to reflect the *T*-caused ST differences, *T* of the samples were recorded after ultrasonication process via an in-house built thermometer. The flowchart summarizing the methodology of this work is given in Fig. 3.

## 3 Results and discussions

This work has a two-fold purpose: (i) ST measurements of ZnO-DW nanofluids dependent on nanoparticle volume fraction, ultrasonication amplitude, and ultrasonication duration; (ii) statistical analyses to quantify these parameters importance on ST and relative ST of ZnO-DW nanofluids. From the literature review presented in Introduction, it is deduced that statistical comparison of parameters effects is required to quantify whether ST or relative ST of nanofluids depend on a material or process variable.

### 3.1 Experimental results

**Fig. 4:**
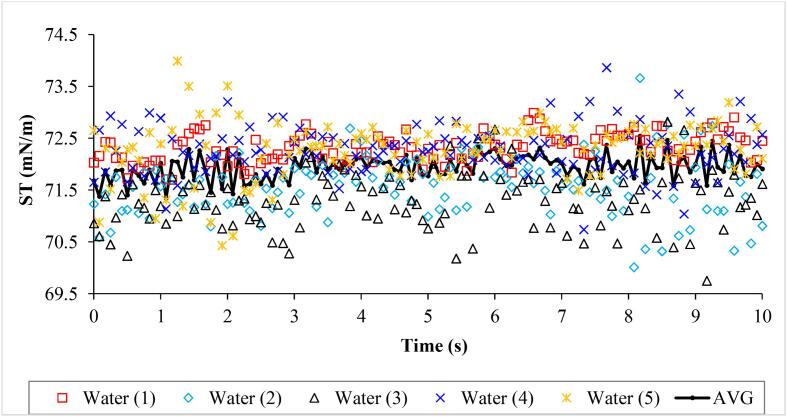
ST results of 5 DW micro-droplets and their arithmetic mean (AVG).

After preparation of ZnO-DW nanofluid samples of 0.05–0.4 vol%, ST measurements were conducted. Before proceeding with ZnO-DW nanofluids ST measurements, the tensiometer was calibrated by performing ST measurements of water droplets. Fig. 4 provides the ST measurements of 5 water droplets over the measurement period.

The ST of water is reported as 72.75 mN/m and 72.76 mN/m [24]. The time-based average of the AVG values as shown in Fig. 4 is 71.94736 mN/m, and its deviation from the values reported by [24] is 1.10329%. After seeing this trend of around 1.1% deviation, ZnO-DW nanofluids ST measurements were performed.

**Fig. 5:**
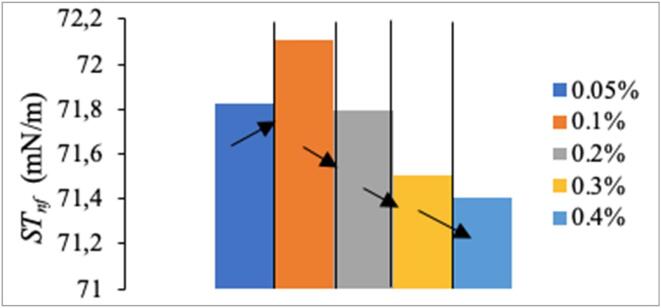
ZnO nanoparticle concentration dependence of *ST_nf_*.

For each sample, ST’s of 5 different micro-drops were measured and averaged to reduce randomized errors. Fig. 5 shows variation of the ST of nanofluids (*ST_nf_*) that were not ultrasonically treated. Hence, Fig. 5 is indicative of how *ST_nf_* changes due to the change of nanoparticle concentration, only. When Fig. 5 is examined, it is seen that the only increase in *ST_nf_* is observed as the nanoparticle concentration doubled from 0.05% to 0.1%. For the other cases, increase of nanoparticle concentration resulted in a continuous decrease in *ST_nf_*.

**Fig. 6:**
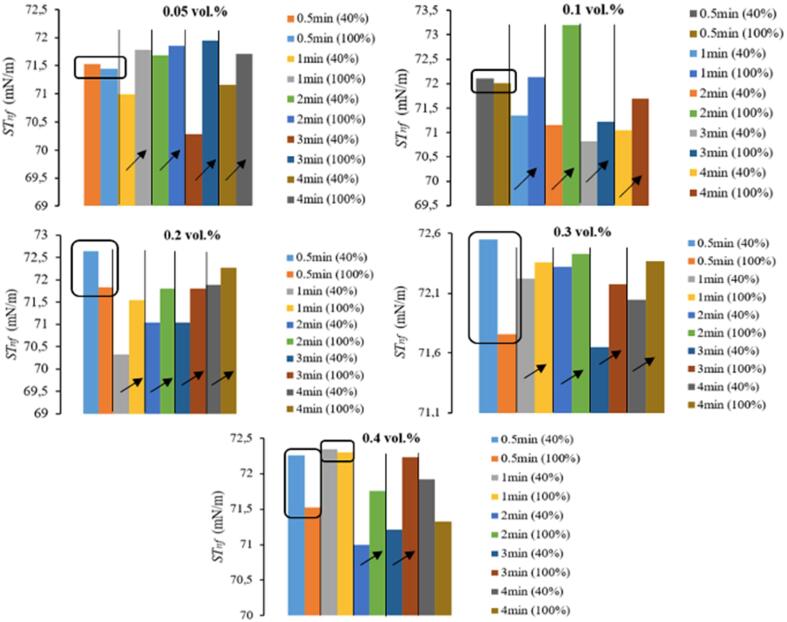
Ultrasonication amplitude (% in the parentheses) and duration dependence of *ST_nf_* of different concentrations of ZnO in DW.

While Fig. 5 depicts the *ST_nf_* of samples that were not treated with ultrasonication, Fig. 6 shows the *ST_nf_* results individually for each nanoparticle fraction (0.05% to 0.4%, respectively), in a comparative manner, dependent on ultrasonication duration and amplitude. The results presented in Fig. 6 include the implicit effect of temperature (*T*), besides the ultrasonication amplitude and duration, which can be considered to contain the overall effect of direct-ultrasonication process. This ultrasonication process affected ST behavior of nanofluids is considered to be of importance since *T* rise after direct ultrasonication is inevitable and expected, making *T* an uncontrolled variable of the experiments. *T* effect on ST of liquids (*ST_bf_*) is well-developed. Since direct ultrasonication resulted in *T* increase of nanofluid samples, especially for longer durations and/or higher amplitude, the *ST_nf_* results reflect *T* variations. For the current case, *T* (*y*) – ultrasonication duration (*x,* 0.5 min to 4 min) are correlated via *y* = 7.4537*x* + 31.807 (*R^2^* = 0.9737) for 40% amplitude, and *y* = 12.34*x* + 38.005 (*R^2^* = 0.9559) for 100% amplitude, while these regression equations are specific to the ultrasonicator and materials including volume of nanofluids, concentration, type and size of nanoparticles studied in this work. Fig. 6 reveals that, for the same concentration, *ST_nf_* of samples ultrasonicated at 100% amplitude were generally greater than those treated at 40% amplitude, except for the 0.5 min processes. This shortest period of ultrasonication provided an initial decrease in *ST_nf_* for 0.05, 0.1, 0.2, 0.3% samples. One exception to this behavior is observed in Fig. 6 for 0.4 vol% sample, which did not reveal an increase of *ST_nf_* for 1 min and 4 min ultrasonication when 100% amplitude is compared to 40% amplitude. On the other hand, when 1–4 min of ultrasonications were evaluated, throughout Fig. 6, 100% amplitude typically provided greater *ST_nf_* compared to those obtained after 40% amplitude for the same duration.

As mentioned by Mahbubul [12], literature studies are not conclusive on nanoparticle concentration dependence of *ST_nf_* due to increases and decreases reported, as a result of increased nanoparticle concentration. Khanafer and Vafai [25] also reported that although nanoparticle concentration effect was not straightforward, *T* had a strong impact on *ST_nf_*. The results in Fig. 5 and Fig. 6 do not reveal a monotonous dependence of either on nanoparticle concentration or ultrasonication parameters. The major reason for these complex dependences is the inherent *T* dependence of *ST_nf_* data due to ultrasonication. In other words, the variations in data could not be attributed solely to nanoparticle concentration, ultrasonication amplitude, and ultrasonication duration, without considering samples *T*. For this purpose, *T* dependence of ST is considered via defining relative ST, as *ST_r_ = ST_nf_ / ST_bf_*. Here, *ST_bf_* stands for the ST of DW, which is calculated at each *T* imposed by horn ultrasonication process, using the following formula from Murshed et al. [11], *ST* = 0.076–0.00017.*T* K^−1^. The *T* values of DW were measured right after they were ultrasonicated at the same conditions as the nanofluid samples were treated. In calculation of *ST_r_,* this was the pursued approach, since the overall approach in nanofluids literature has been to compare nanofluid samples with their base fluid counterparts to see if utilization of nanofluids would bring advantages or challenges, and to make ultrasonication process the constant treatment/effect in this work

**Fig. 7:**
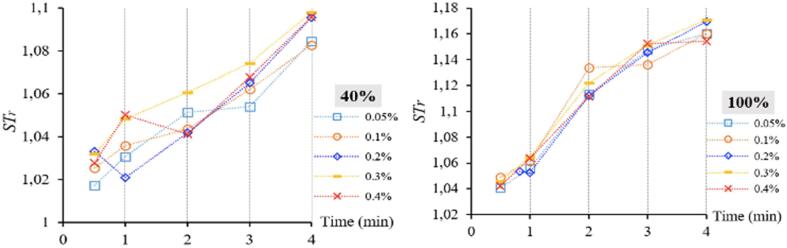
Ultrasonication duration, amplitude and ZnO concentration (vol.%) dependence of *ST_r_*.

Fig. 7 depicts the *ST_r_* trends of the studied nanofluids with respect to ultrasonication process. Since *T* effect is extricated from the ST behavior, *ST_r_* trends are clearer and more relatable. The first observation from Fig. 7 would be the typical *ST_nf_* increase from *ST_bf_* as the ultrasonication process is applied for longer durations/higher amplitude. In other words, ZnO-DW nanofluids of this work mostly showed a positive correlation of *ST_r_* with ultrasonication processes.

**Fig. 8:**
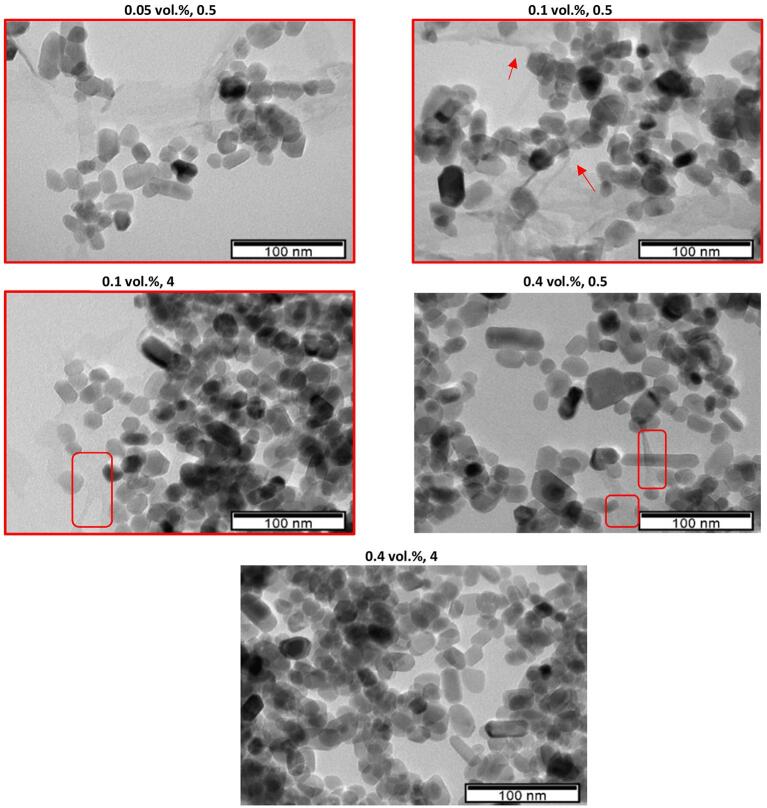
TEM images of ZnO-DW nanofluids at different concentrations and ultrasonicated at 40% amplitude.

**Fig. 9:**
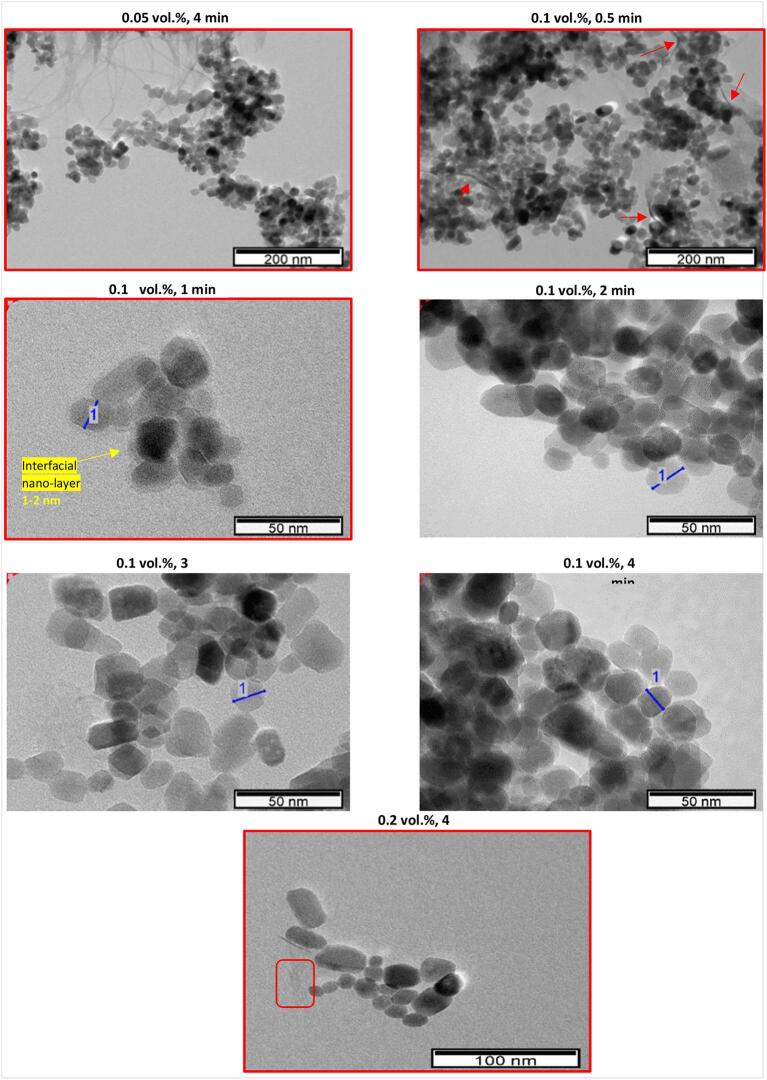
TEM images of ZnO-DW nanofluids at different concentrations and ultrasonicated at 100% amplitude.

It is seen that neither *ST_nf_* nor *ST_r_* is strongly correlated to nanoparticle fraction. On the other hand, surface behavior, similar to thermophysical properties, is related to solid particle size and cluster formation. Electron microscopy imaging is used to capture how particles presence vary as the preparation conditions (e.g., nanoparticle concentration, ultrasonication amplitude, ultrasonication duration) change. TEM images obtained from samples are given in Fig. 1 (for the samples that were not ultrasonicated) and Fig. 8, Fig. 9 (for the ultrasonicated samples).

Fig. 8 and Fig. 9 shows the TEM images from selected samples ultrasonicated respectively at 40% and 100% amplitude. The TEM imaging of the samples were taken within a few days after the samples were prepared. In Fig. 9, nanoparticles in 0.1 vol% suspension ultrasonicated for 4 min appeared in a more aggregated form when compared to those in 0.1 vol% sample ultrasonicated for 3 min. Such a result might have arisen from the fact that there was an optimum ultrasonication duration (3 min) for lower polydispersity, beyond which ultrasonication promotes cluster formation by excessively increasing kinetic energy of nanoparticles at this highest (100%) amplitude. On the other hand, it should be noted that particles appearing in agglomerated state may not be directly indicative of how nanoparticles would actually be dispersed in the nanofluid, and images showing more/less crowded areas do not necessarily correspond to more/less clustered conditions.

Another feature seen in the TEM images, both for 40% and 100% is nano-sized layers. A 1–2 nm thick inter-facial layer is observed in the TEM image of the 0.1 vol% sample that was ultrasonicated for 1 min at 100% amplitude. The thickness of this nano-layer lies at the sensitivity limit of the TEM, therefore absence of a nano-film in the micrographs of other samples may be due to either absence of it or device being unable to detect it, for thicknesses below 1–2 nm. In the literature, an interfacial layer, as an ordered layer of liquid molecules around the particle-base fluid interface ([26], [27]) has been mentioned. Generally appearing as a nanometer-thick component, it consists of fluid molecules bonded/oriented at the interface of nanoparticle and base fluid, exhibiting properties different than both [28]. This component can cause alterations in nanofluids surface behavior due to higher surface-to-volume ratio of nanoparticles, and probably by increasing the effective nanoparticle volume fraction [26].

Murshed et al. [28] used a model that was proposed by Hashimoto et al. [29] for predicting the thickness of interfacial nanolayer (*t*) of nanofluids:

(1) $$t=\sqrt{2\pi }\sigma$$

In Eq. (1), σ characterizes the diffuseness of the boundary [29]. Greenwood et al. [30] stated that the adsorbed layer thickness on a particle decreased with particle size, in their work concerned with polystyrene and PMMA particles with adsorbed stabilizing copolymer as the interfacial layer. It should be noted that although interfacial layers are formed on micro- and nanoparticles, its effect is much more pronounced for nanoparticles due to their increased specific surface area [27].

Morphologically, the nano-layer observed in the TEM image of 0.1 vol% sample ultrasonicated for 1 min at 100% amplitude is a typical interfacial layer in terms of the way it frames the particles (i.e., from the perimeters). On the other hand, other nano-films are observed from the TEM images of 0.05 vol% ultrasonicated for 4 min, 0.1 vol% ultrasonicated for 30 s, 0.2 vol% ultrasonicated for 4 min in Fig. 8; and 0.05 vol% ultrasonicated for 30 s, 0.1 vol% ultrasonicated at 30 s, 0.1 vol% ultrasonicated for 4 min, 0.4 vol% ultrasonicated for 0.5 min in Fig. 8. During TEM imaging, the chemical composition of this nano-film was checked via EDS (by spotting on the nano-film) which revealed the presence of Zn, O and Cu (from Cu grid used for EDS analysis of sample) species (as in Fig. 2). It was also free of common and organic contaminants, as well as the materials present nearby where these nanofluids were stored. During this work, to avoid contamination from the ultrasonic tip and apparatus used for transfer of nanofluids as much as possible, the tip and pipettes were cleaned before and after the use of these equipment. These nano-films are morphologically different from the interfacial layer as shown in Fig. 9 for 0.1 vol% sample ultrasonicated for 1 min, as (i) they cover the nanoparticle and clusters like a “blanket” and they fold (emphasized with arrows in Fig. 8 and Fig. 9), (ii) they extend away from the nanoparticle clusters (typically the TEM image of 0.05 vol% ultrasonicated for 4 min, in Fig. 8) by at least several times greater length of individual nanoparticle diameter, and connect to the neighbor nanoparticle cluster nearby, (iii) they appear as elastic nano-paths linking smaller parts of clusters or even individual particles in the neighboring nanoparticle clusters, and (iv) its Zn component confirmed via EDS analyses suggests that it may be an intermediate or more solid-like layer, unlike an interfacial layer. Therefore, this transparent and very thin component of the colloidal system is named a “nano-film”, as a component formed due to ZnO-DW interaction that is structurally different from an interfacial layer and is expected to influence the ST of the nanofluid by altering the cohesive and adhesive forces among the molecules in the base fluid. Okubo [31] demonstrated that ST of colloidal suspensions of polystyrene and silica particles in water was correlated to adsorption of ions at the particle surface, and when electrical double layers (EDLs) were expanded, colloidal crystals could be formed. Although formation of such ordered structures was not expected for those polystyrene and silica spheres of smaller than 50 nm [31], particles of different materials may encounter such crystal structure even at smaller size as the ZnO particle (around 15–20 nm) of the current work. In addition, EDLs are inherent features of nanoparticles dispersions and they can impact the colloidal surfaces, potentially resulting in changes in *ST_nf_*. With the light of the discussion on the possible formation mechanisms and roles of the nano-sized component, more extensive research is needed to confirm the composition and structure as well as its form as a nano-film linking the nanoparticles together.

The observed nano-films were formed when the nanoparticle concentration is rather low, i.e., 0.05%, 0.1%, and 0.2%. For these concentrations, ultrasonication for shorter durations is mostly favorable in nano-film formation, except for the case of 0.1 vol% sample ultrasonicated at 40% amplitude for 4 min. It can be argued for this exception that an amplitude of 40% was not sufficient to damage this nano-film that is present for the case of low concentrations and low process durations. Furthermore, it may directly affect the *ST_nf_* and *ST_r_*.

### 3.2 Statistical assessment

As statistical assessments provide valuable information on whether variables differ from each other in a statistically significant way, or whether they are correlated to one another with a weak, intermediate, or strong relation, they strengthen interpretation of experimental results. The experiments were planned by deciding on the independent variables, independent variables levels (categories), and the number of repetitions. This approach is named the design of experiments (DOE) and upon proper data collection and adequate number of data points, it is possible to investigate the effects of independent variables on the dependent variable(s) (e.g., as in [32]). In addition to such statistical work, soft-computing based analyses are also of importance when interpreting nanofluid data. Such methods include artificial neural networks, fuzzy-logic, adaptive neuro-fuzzy interference system (ANFIS) to name a few ([33], [34], [35]).

In this work, dependent variables are *ST_nf_* and *ST_r_* while independent variables are nanoparticle concentration, ultrasonication amplitude, and ultrasonication duration. Table 1 elaborates on the dependent and independent variables of this work.

**Table 1:** Experiment parameters and their levels.

Independent variables	Values	Number of levels
Nanoparticle concentration (%)	0.05, 0.1, 0.2, 0.3, 0.4	5
Ultrasonication duration (min)	0.5, 1, 2, 3, 4	5
Ultrasonication amplitude (%)	40, 100	2
Dependent variables	*ST_nf_, ST_r_*	

For each experiment at a specified nanoparticle concentration, ultrasonication duration, and ultrasonication amplitude, *ST_nf_* data were collected from 5 different droplets, and from these data, *ST_r_* were calculated. The experiment set involves 250 data points for *ST_nf_* and *ST_r_*. Based upon this experimental database, the following comparisons are made.

#### 3.2.1 Variation in *ST_nf_* and *ST_r_* with ultrasonication amplitude

Before performing a statistical analysis, the variables distributions are evaluated. As a rule of thumb, one proceeds with parametric tests if distributions are normal, while nonparametric tests were regarded distribution freer tests [36], [37] and can generally be used without assuming a specific distribution. An initial examination of variables showed that the distributions in *ST_nf_* and *ST_r_* within the levels of amplitude variable is not normal. Amplitude has two discrete levels (40% and 100%). Here the aim is to evaluate whether there is a statistically significant difference in *ST_nf_* and *ST_r_* as amplitude’s value changes. For this purpose, Mann Whitney *U* test, which is a nonparametric test allowing to test one set of scores tending to be differing than another set [37], [38] is used in this study. The hypotheses to be tested for Mann-Whitney *U* test is as follows (here the variables names are generalized, and hypotheses are not repeated in the following sections): H_0_ (null hypothesis): The two populations are equal. H_a_ (alternative hypothesis): The two populations are not equal. Based on the results, if the significance level (*p*) ≤ 0.05, H_0_ is rejected and H_a_ is accepted. Otherwise, if *p* > 0.05 H_0_ is accepted and H_a_ is rejected.

Based on the results in Table 2, at a significance level (*p*) of 0.05 (i.e., a confidence level of 95%), both *ST_nf_* and *ST_r_* values differ significantly when amplitude varies from 40% to 100% (and vice versa), as *p* ≤ 0.05 for both cases.

**Table 2:** Mann Whitney *U* test results for *ST_nf_* and *ST_r_* with ultrasonication amplitude change.

Test Statistics^a^		
	*ST_nf_*	*ST_r_*
Mann-Whitney U	5395.000	2929.000
Wilcoxon W	13270.000	10804.000
Z	−4.229	−8.542
*p* (Asymp. Sig. (2-tailed))	0.000024	<0.0001
a. Grouping Variable: Coded_amplitude		
Test Statistics^a^		

The results in Table 2 leads to the conclusion that based upon this dataset, when examining ZnO-DW nanofluids ST and relative ST, if ultrasonication amplitude cannot be kept constant, its effect would reflect both in *ST_nf_* and *ST_r_* data. Another important point is to examine how *ST_nf_* and *ST_r_* data of this work are correlated to ultrasonication amplitude. This is indicative of how *ST_nf_* and *ST_r_* vary as amplitude changes, and what is the extent of the relationship. Spearman’s rho is a non-parametric correlation coefficient [37] used since the distributions are not normal (as in the selection of Mann-Whitney *U* test). Table 3 shows the correlation analysis results of *ST_nf_* and amplitude, with *ST_r_* and amplitude.

**Table 3:** Correlation analysis on the relation between *ST_nf_ -*amplitude and *ST_r_-*amplitude.

Correlations		*ST_nf_*	Amplitude
*ST_nf_*	Correlation Coefficient (Spearman's rho)	1.000	0.268^**^
	*p* (Sig. (2-tailed))	.	0.000017
	N	250	250
Amplitude	Correlation Coefficient	0.268^**^	1.000
	*p* (Sig. (2-tailed))	0.000017	.
	N	250	250
		*ST_r_*	Amplitude
*ST_r_*	Correlation Coefficient (Spearman's rho)	1.000	0.541^**^
	*p* (Sig. (2-tailed))	.	0.000
	N	250	250
Amplitude	Correlation Coefficient	0.541^**^	1.000
	*p* (Sig. (2-tailed))	<0.0001	.
	N	250	250
**. Correlation is significant at the 0.01 level (2-tailed).			

Similar to the Mann Whitney *U* test (as given in Table 2), correlation analysis results can be interpreted based on the *p* value, while this time correlation coefficients value is also important, as it indicates the extent of the relationship between the variables. Correlation coefficient can have values between 0 and 1. Gerber and Finn [39] summarized the correlation coefficient-correlation ranges as 0–0.30 weak, 0.31–0.60 moderate, and > 0.60 strong. The hypotheses to be tested for correlation analysis is as follows (here the variables names are generalized, and hypotheses are not repeated in the following sections): H_0_ (null hypothesis): The correlation coefficient between two variables is zero. H_a_ (alternative hypothesis): The correlation coefficient between two variables is nonzero. Based on the results, if the significance level (*p*) ≤ 0.05, H_0_ is rejected and H_a_ is accepted. Otherwise, if *p* > 0.05 H_0_ is accepted and H_a_ is rejected. Based on this information, both the *ST_nf_* and *ST_r_* are correlated to the amplitude (*p* < 0.05). While *ST_nf_ -*amplitude correlation is weak (0.268 < 0.30), *ST_r_-*amplitude correlation is moderate. This supports the previous experimental data-based conclusion that amplitude- *ST_r_* relation is easier to interpret than that between *ST_nf_ -*amplitude.

#### 3.2.2 Variation in *ST_nf_* and *ST_r_* with nanoparticle concentration

As shown in Table 1, nanoparticle concentration has 5 levels. In order to assess the effect of nanoparticle concentration, the minimum and maximum levels (corresponding respectively to 0.05 vol% and 0.4 vol%) are considered. *ST_nf_* and *ST_r_* within the levels of nanoparticle concentration is not normal, and comparisons are made based upon the Mann Whitney *U* test.

The outputs listed in Table 4 show that *ST_nf_* data changes significantly as the nanoparticle concentration changes from 0.05 to 0.4 vol% (*p* ≤ 0.05), while *ST_r_* results show the opposite by *p* > 0.05, i.e., change of nanoparticle concentration from 0.05 to 0.4 vol% do not cause a statistically significant variation in *ST_r_* at a significance level of 0.05. Table 5 presents correlation analysis results for the *ST_nf_ -*nanoparticle concentration and *ST_r_-*nanoparticle concentration.

**Table 4:** Mann Whitney *U* test results for *ST_nf_* and *ST_r_* with nanoparticle concentration.

Test Statistics^a^		
	*ST_nf_*	*ST_r_*
Mann-Whitney U	825.000	1146.000
Wilcoxon W	2100.000	2421.000
Z	−2.930	-0.717
*p* (Asymp. Sig. (2-tailed))	0.003	0.473
a. Grouping Variable: Coded_vol. fraction		
Test Statistics^a^

**Table 5:** Correlation analysis on the relation between *ST_nf_ -*volume fraction and *ST_r_ -* volume fraction.

Correlations		ST_nf_	Vol. fraction
*ST_nf_*	Correlation Coefficient (Spearman's rho)	1.000	0.295^**^
	*p* (Sig. (2-tailed))	.	0.000002
	N	250	250
Vol.fraction	Correlation Coefficient	0.295^**^	1.000
	*p* (Sig. (2-tailed))	0.000002	.
	N	250	250
		ST_r_	Volfraction
*ST_r_*	Correlation Coefficient (Spearman's rho)	1.000	0.080
	*p* (Sig. (2-tailed))	.	0.205
	N	250	250
Vol. fraction	Correlation Coefficient	0.080	1.000
	*p* (Sig. (2-tailed))	0.205	.
	N	250	250
**. Correlation is significant at the 0.01 level (2-tailed).			

Correlation analysis reveal that there is a correlation between *ST_nf_* and volume fraction (*p* ≤ 0.05) while *ST_r_*-volume fraction correlation is not significant (*p* > 0.05). Supporting these results, it is seen that the correlation between *ST_nf_ -*volume fraction is weak to moderate.

#### 3.2.3 Variation in *ST_nf_* and *ST_r_* with ultrasonication duration

Similar to nanoparticle concentration, ultrasonication duration consists of 5 discrete levels, as shown in Table 1. In order to assess the effect of ultrasonication duration, the minimum and maximum levels (corresponding respectively to 0.5 min and 4 min) are considered in this analysis. Normality distribution check showed that *ST_nf_* distributed normally within the levels of ultrasonication duration, while *ST_r_* did not show normal distribution. In this case, the effect of ultrasonication duration on *ST_nf_* can be evaluated via one-way ANOVA (Analysis Of Variance) test, while the change in *ST_r_* as ultrasonication duration varies from 0.5 to 4 min is evaluated via the Mann Whitney *U* test. On the other hand, one-way ANOVA requires the homogeneity of variances [37], which is not satisfied by *ST_nf_* data. Therefore, both the *ST_nf_* – ultrasonication duration and *ST_r_* -ultrasonication duration variations are evaluated via the Mann Whitney *U* test. The results are shown in Table 6.

**Table 6:** Mann Whitney *U* test results for *ST_nf_* and *ST_r_* variation as ultrasonication duration changes.

Test Statistics^a^		
	*ST_nf_*	*ST_r_*
Mann-Whitney U	1066.000	0.000
Wilcoxon W	2341.000	1275.000
Z	−1.268	−8.617
*p* (Asymp. Sig. (2-tailed))	0.205	<0.0001
a. Grouping Variable: Coded_duration		

The results show that, as the ultrasonication duration changes from 0.5 to 4 min (and vice versa) *ST_nf_* values do not show a statistically significant difference (*p* > 0.05), whereas the same conditions result in a statistically significant variation in *ST_r_* data. Correlation analysis revealing the relation between *ST_nf_* - duration and *ST_r_* - duration resulted in the data shown in Table 7. Here, Pearson correlation coefficient is used for *ST_nf_* -ultrasonication correlation. Pearson correlation coefficient assumes binormal distribution [40]. Since *ST_nf_* has a normal distribution within the levels of duration, this coefficient should be used and interpreted. On the other hand, since *ST_r_* does not have a normal distribution, use of Spearman’s rho is a proper choice to interpret correlation.

**Table 7:** Correlation analysis on the relation between *ST_nf_ -*ultrasonication duration and *ST_r_ -* ultrasonication duration.

Correlations		*ST_nf_*	Duration
*ST_nf_*	Pearson Correlation	1	-0.148*
	*p*(Sig. (2-tailed))		0.020
	*N*	250	250
Duration	Pearson Correlation	-0.148*	1
	*p*(Sig. (2-tailed))	0.020	
	*N*	250	250
		*ST_r_*	Duration
*ST_r_*	Correlation Coefficient (Spearman's rho)	1.000	0.780^**^
	*p*(Sig. (2-tailed))	.	<0.0001
	*N*	250	250
Duration	Correlation Coefficient	0.780^**^	1.000
	*p*(Sig. (2-tailed))	<0.0001	.
	*N*	250	250

Based on the results summarized in Table 7, it is observed that both *ST_nf_* and *ST_r_* are correlated to ultrasonication duration (*p* < 0.05). This is supported by the correlation coefficients so that a strong correlation between *ST_r_* and ultrasonication duration is present (0.780 > 0.70) while the correlation between *ST_nf_* and ultrasonication duration is weak (0.148 < 0.30).

In summary, the results of Mann-Whitney U tests and correlation analyses showed the significance or insignificance of the differences in *ST_nf_* and *ST_r_* upon the changes in ultrasonication amplitude, nanoparticle concentration, and ultrasonication duration. When amplitude varies, there is a statistically significant difference in *ST_nf_* and *ST_r_* values. Variation of nanoparticle concentration causes a significant difference in *ST_nf_* while the difference is insignificant on *ST_r_*. The difference *ST_nf_* by variation of ultrasonication durations is not significant, while the difference is significant with *ST_r_*. Correlation analysis results showed that *ST_nf_ -*amplitude correlation is weak while *ST_r_-*amplitude correlation is moderate. The correlation between *ST_nf_ -*volume fraction is weak to moderate. S*T_nf_* and duration are weakly correlated while there is a strong correlation between *ST_r_ -*duration. Investigating parameters effects and correlations are important, since it brings the opportunity to tune the ST as the application necessitates.

## 4 Conclusions

Based on the findings of this work on ZnO-DW nanofluids, the following conclusions are made:

• Ultrasonication is an essential process for proper dispersion of nanoparticles in base fluid and stability, as well as for optimum properties for real-world applications. However, there is a clear lack of understanding on the effect of ultrasonication parameters on certain properties and applications. This study experimentally investigates ultrasonically-tuned ST of ZnO nanofluids for potential microfluidics application.
• This work is the first report of ST – ultrasonication parameters relation, and it may shed a light on surface properties of ZnO-DW nanofluids for their potential applications particularly in microfluidic systems.
• As a consequence of direct ultrasonication, *T* rise is a widely observed phenomenon, especially for small sample volumes. This is mostly a trade-off between better stability expectance and sample *T* rise (and even evaporation) as the ultrasonication intensifies. On the other hand, as stable ZnO-DW nanofluids require ultrasonication, it is important to keep ultrasonication effect unbiased while observing its impacts on ST. In this case, *T* increase and decrease effects are present on ST values, making other comparisons more complicated, unless the effect of *T* is isolated. For this purpose, this work demonstrates using relative ST as a comparison merit between *ST_nf_* and *ST_bf_* when ultrasonication is a must.
• ST and nanofluid/process parameter relations mostly were not well-developed, and quantifying parameters effects and relations between variables is a necessary task. The ultrasonication process history including the amplitude, power, duration, ultrasonication mode (continuous or pulsed), as well as the type of ultrasonication are important factors to affect the nanofluids structurally and cause variations in thermophysical and electrokinetic properties including surface tension.
• Results of statistical analyses (mainly Mann-Whitney U tests) provide more insight on experimental data, showing the differences in *ST_nf_* and *ST_r_* upon variations in ultrasonication amplitude, ultrasonication duration, and nanoparticle volume fraction.
• Correlation analysis showed that use of *ST_r_* rather than *ST_nf_* results in amplified effect of amplitude, by the correlation coefficients of 0.286 and 0.541, respectively. This means that *ST_r_* – ultrasonication amplitude correlation is stronger than that of *ST_nf_* – ultrasonication amplitude. Effects augmentation is much more pronounced going from *ST_nf_* to *ST_r_* when the independent variable is ultrasonication duration. When *ST_nf_* and duration are weakly correlated, *ST_r_* and duration has a strong correlation.
• At this point it’s not well-understood how presence of the nano-film observed by TEM imaging can affect the ST and other properties. Thus, effect of such nano-film on surface behavior and wetting of nanofluids should be investigated.

## CRediT authorship contribution statement

**Elif Begum Elcioglu:** Investigation, Conceptualization, Formal analysis, Visualization, Writing - original draft. **S.M. Sohel Murshed:** Supervision, Conceptualization, Methodology, Resources, Writing - review & editing.

## Declaration of Competing Interest

The authors declare that they have no known competing financial interests or personal relationships that could have appeared to influence the work reported in this paper.
